# A curated global dataset of social contact between diverse language communities

**DOI:** 10.1038/s41597-025-06192-1

**Published:** 2025-12-11

**Authors:** Eri Kashima, Francesca Di Garbo, Oona Raatikainen, Robert Forkel, Rosnátaly Avelino, Sacha Beck, Anna Berge, Ana Blanco Pena, Ross Bowden, Nicolás Brid, Joseph M. Brincat, María Belén Carpio, Alexander Cobbinah, Paola Cúneo, Deginet Wotango Doyiso, Anne-Maria Fehn, Saloumeh Gholami, Arun Ghosh, Hannah Gibson, Elizabeth Hall, Katja Hannß, Hannah Haynie, Jerry J. Jacka, Mathias Jenny, Richard Kowalik, Sonal Kulkarni-Joshi, Maarten Mous, Marcela Mendoza, Cristina Messineo, Francesca Romana Moro, Hank Nater, Michelle Ocasio, Bruno Olsson, Ana María Ospina Bozzi, Agustina Paredes, Admire Phiri, Nicolas Quint, Erika Sandman, Dineke Schokkin, Ruth Singer, Ellen Smith-Dennis, Lameen Souag, Yunus Sulistyono, Yvonne Treis, Matthias Urban, Jill Vaughan, Georg Ziegelmeyer, Veronika Zikmundová, Ricardo Napoleão de Souza, Kaius Sinnemäki

**Affiliations:** 1 University of Helsinki, Department of General Linguistics, Helsinki, Finland; 2 The Australian National University, School of Culture, History, and Language, Canberra, Australia; 3 Aix-Marseille Univ. - CNRS LPL UMR 7309, Aix-en-Provence, France; 4 Independent Researcher, Helsinki, Finland; 5 Max Planck Institute for Evolutionary Anthropology, Department of Linguistic and Cultural Evolution, Leipzig, Germany; 6 Secretaría de Ciencia, Humanidades, Tecnología e Innovación (SECIHTI) - Universidad Nacional Autónoma de México, Instituto de Investigaciones Filológicas, Ciudad de México, México; 7 Independent Researcher, Brussels, Belgium; 8 University of Alaska Fairbanks, Alaska Native Language Center, Fairbanks, USA; 9 Instituto de Lingüística, Universidad de Buenos Aires, Buenos Aires, Argentina; 10 Independent Researcher, Melbourne, Australia; 11 L-Università ta’ Malta, Faculty of Arts, Msida, Malta; 12 Consejo Nacional de Investigaciones Científicas y Técnicas (CONICET), Buenos Aires, Argentina; 13 Universidad Nacional del Nordeste, Corrientes, Argentina; 14 Universidade de São Paulo, São Paulo, Brazil; 15 Independent Researcher, Cologne, Germany; 16 BIOPOLIS-CIBIO: Research Centre in Biodiversity and Genetic Resources - InBIO Associate Laboratory, Vairão, Portugal; 17 University of Cambridge, Faculty of Asian and Middle Eastern Studies, Cambridge, United Kingdom; 18 University of Burdwan, Bardhaman, India; 19 Georg-August-Universität Göttingen, Göttingen, Germany; 20 University of Essex, Department of Language and Linguistics, Colchester, United Kingdom; 21 SIL Global, Dallas, USA; 22 University of Cologne, Department of Linguistics, Cologne, Germany; 23 University of Colorado, Department of Linguistics, Boulder, USA; 24 University of Colorado, Department of Anthropology, Boulder, USA; 25 Chiang Mai University, Faculty of Humanities, Chang Wat Chiang Mai, Thailand; 26 UiT The Arctic University of Norway, Department of Language and Culture, Tromsø, Norway; 27 Deccan College, Post-Graduate and Research Institute, Pune, India; 28 Leiden University, Centre for Linguistics, Leiden, Netherlands; 29 University of Oregon, Department of Anthropology, Eugene, USA; 30 University of Naples L’Orientale, Department of Asia, Africa and Mediterranean, Napoli, Italy; 31 Independent Researher, Alberta, Canada; 32 Valdosta State University, Modern and Classical Languages, Valdosta, USA; 33 University of Regensburg, Department of General Comparative Linguistics, Regensburg, Germany; 34 Departamento de Lingüística, Universidad Nacional de Colombia, Bogotá, Colombia; 35 University of the Free State, Department of Linguistics and Language Practice, Bloemfontein, South Africa; 36 University of Porto, Faculty of Arts and Humanities, Porto, Portugal; 37 CNRS LLACAN - UMR 8135 (CNRS / INALCO / EPHE), Villejuif, France; 38 Independent Researcher, Christchurch, New Zealand; 39 University of Melbourne, School of Languages and Linguistics, Parkville, Australia; 40 University of Newcastle, Callaghan, Australia; 41 CNRS LACITO - UMR 7107 (CNRS - Sorbonne Nouvelle - INALCO), Villejuif, France; 42 Indonesian Language and Literature, Muhammadiyah University of Surakarta, Surakarta, Indonesia; 43 DDL UMR 5596, CNRS & Université Lumière Lyon 2, Lyon, France; 44 Monash University, School of Languages, Literatures, Cultures and Linguistics, Clayton, Australia; 45 University of Vienna, Institute of African Studies, Wien, Austria; 46 Charles University, Institute of Asian Studies, Prague, Czech Republic; 47 Hanken School of Economics, Helsinki, Finland

**Keywords:** Culture, Social anthropology

## Abstract

The GramAdapt Social Contact Dataset is a curated dataset of 34 language pairs with qualitative and quantifiable data on social interaction and aspects of societal multilingualism. The language pairs were sampled globally to represent the world’s linguistic diversity. The dataset can be used to interrogate the social dimensions of language contact independently or in conjunction with appropriate linguistic data. The data were collected by distributing a questionnaire to experts who have experience with either one or both of the language communities of a pair. The data represent subjective expert assessments based on choices from predetermined answers which can be quantified. Authors 1, 2 and 3 manually checked the response to identify possible misjudgments or misunderstandings. This results in a dataset containing 13,493 data points. This dataset is a first of its kind in the field of linguistics, built upon wide findings from sociolinguistics, historical linguistics, psycholinguistics, and linguistic anthropology.

## Background & Summary

Language contact research investigates linguistic change resulting from bilingual and multilingual speakers. Linguistic change ranges from simple word borrowing (e.g., *pajama* from Persian via Urdu into English) to grammatical restructuring such as changes in word order tendencies (e.g., Finno-Ugric Finnish changing the dominant word order to Subject-Verb-Object through contact with Germanic languages). Hypotheses concerning language contact outcomes have focused on the linguistic elements that are likely to be lost or gained through contact, such as the loss of dual pronouns (e.g. “you” singular vs. “you-two” dual) when contact is with one language that lacks these types of distinctions (e.g., Eskimo Aleut in contact with Eyak). Language contact outcomes thus fall into the broader area of language adaptation studies where languages are proposed to adapt to their environments. We view multilingual speakers and their social, cultural and historical contexts (or language ecologies) as environments for linguistic adaptation.

Prior global studies of language contact and its social dimensions have investigated specific kinds of language contact phenomena. For example the *Atlas of Pidgin and Creole Language Structures*^1^ targets 76 pidgins and creoles over 28 socio-demographic variables. Another example, the *Expanded Graded Intergenerational Disruption Scale*^2,3^ considers a number of social contexts to classify the threat of language change and death in contact scenarios. The results of these studies are relevant to the specific contact scenarios under investigation, but cannot be generalised to other kinds of communication, such as stable multilingualism in urban or rural areas. That is, they cannot show us whether there are general tendencies in language contact.

**Fig. 1:**
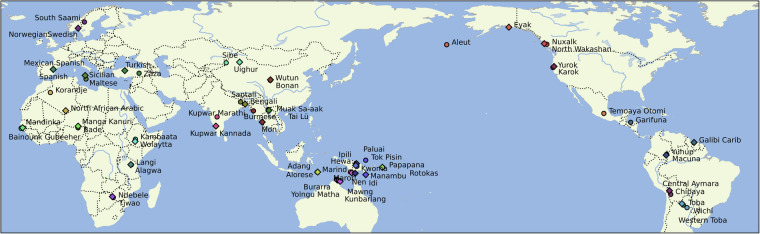
Geographic distribution of the 34 language pairs. Focus languages are shown with a circle marker, neighbour languages with a diamond.

The GramAdapt Social Contact Questionnaire provides two points of differentiation. The first is that the represented contact scenarios are diverse; from small indigenous languages (e.g., contact between Nen and Idi in Papua New Guinea), to colonial contact (e.g., contact between Toba and Spanish in the Grand Chaco, South America). The languages were sampled to represent the linguistic diversity of the world controlling for language families and regions. The spatial distribution of these languages is shown in Fig. 1. The second point of differentiation is the breadth of interactional and language ecological variables covered. These variables were chosen to apply to as many contact contexts as possible, and were built upon explanatory factors that have been identified as relevant to language change.

The resulting dataset contains 13,493 data points representing 34 pairs of languages in contact. For each pair, the responses provide qualitative and quantifiable data on social interaction between peoples of the two language groups,as well as variables related to the bilingual language ecology.

The data were collected by distributing a questionnaire to experts who have experience with either one or both of the language communities of a pair. The data represent subjective expert assessments based on choices from predetermined answers. The questionnaire was designed by authors 1 and 2, with input from authors 3, 49 and 50 as part of the GramAdapt Project (see below), specifically to produce this dataset. Author 4 was involved in the technical validation and implementation of the CLDF architecture. Authors 5-48 were expert respondents to the questionnaire, and are authors of this paper.

The dataset and methodology were created as part of the GramAdapt Project funded by the European Union’s Horizon 2020 research and innovation program. The dataset was created with the intention of connecting with linguistic data to investigate possible relationships between linguistic structure and sociolinguistic factors. The dataset can, nonetheless, be used independently to gain an overview of various contact scenarios and to investigate patterns and relations across the language pairs.

## Methods

### Choice of language communities and sampling method

The dataset consists of 34 pairs of language communities in contact. Each pair consists of what are designated the *Focus language* and *Neighbour language*. The Focus language is, as the term suggests, the focal language community whose perspective is taken. The Neighbour language is the specific language community whose contact relationship with the Focus language is investigated.

The pairs of communities are located geographically close to each other, but the languages are not related by common descent, e.g., South Saami, a language of the Finno-Ugric family, and Norwegian Swedish of the Indo-European family. The criterion of genealogical unrelatedness was included for the benefit of answering linguistic questions when this dataset is linked with linguistic data. In order to assess the probability of any given linguistic feature being the result of contact in the Focus language of each contact pair, a third unit of comparison is added to the picture. This is the Benchmark Language, a close relative of the Focus Language, which is not in contact with either the Focus nor the Neighbor language community. Benchmark languages are only used for the purpose of linguistic comparison and play no role in the description of social interactions between Focus and Neighbor communities, which is why we do not discuss them further in this paper^4^.

The 34 contact pairs are the units of comparison to probe the diversity of contact scenarios worldwide. One contact pair – Sicilian and Maltese – was considered to have gone through two extended, yet distinct phases of contact, where historical documentation is available to fill out the questionnaire. Thus, this contact pair resulted in two sets of responses to the questionnaire.

Each language pair is assigned a numerical ID for brevity in the dataset. For example, South Saami and Norwegian-Swedish is set30, and Sicilian and Maltese is set06a for the present and set06b for the past. The numbers are from 01-34 with no meaning assigned to the order.

Contact pairs were selected in three steps.

1. 24 regions of interest world-wide were identified. These regions are based on the AUTOTYP^5^ database’s areal classification, and provide a grid for focussing on larger continental areas. We established that two candidate contact pairs should be sought for each of the 24 AUTOTYP areas. For instance the two sets sampled for Southern Africa are Langi and Alagwa (set04) and Ndbele and Tjwao (set07).
2. Contact scenarios for each of the AUTOTYP areas were identified by consulting linguistics literature. This literature encompassed large surveys of linguistic areas and their historical/anthropological make-up as well as more detailed descriptions of individual communities and contact scenarios^6,7^. Existing information on types of contact scenarios and their linguistic outcomes played no role in the selection process.
3. Lists of possible collaborators were identified for each of the preselected contact scenarios. This step is a crucial part of the methodology due to the availability of scientific knowledge concerning many of the sampled languages. The list of experts were updated based on the responses that we received to our initial survey, which aimed at introducing the questionnaire to potential respondents and, in some cases, to solicit feedback concerning the choice of languages. When possible, we attempted to establish contact with researchers that are also members of the communities under study, as is the case for set07 (Ndebele and Tjawo, Southern Africa) or, at least in part, set04 (Rangi and Alagwa, Southern Africa). In the majority of cases, the researchers who agreed to complete the questionnaire have long-term experience with the communities under study, but are community outsiders. Further details on how these experts were selected is outlined in the section below.

In sum, the selection procedure for this dataset was inspired by standard desiderata in the language sampling literature: maximising the representativeness and independence of data points by introducing some form of systematic genealogical and areal control^8,9^. The languages of each contact pair belong to different language families and are selected using the AUTOTYP areal classification to narrow the focus in wider continental areas. The geographic distribution of the sampled contact pairs is shown in Fig. 1.

### Selection of expert collaborator respondents

The questionnaire was filled out by 45 expert collaborators (authors 5–48). The expert collaborators (henceforth “respondents”) were approached as academic collaborators, not as volunteers in an experiment. This process of approaching expert collaborators was screened and approved by the European Research Council Executive Agency (Ethics Review) before the launch of the project. All 45 respondents of the questionnaire are authors of the manuscript.

The respondents were chosen from the field of linguistics and anthropology, based on their publication output and fieldwork experience with either or both Focus and Neighbour language communities. Most respondents are linguists (n = 44/47), and are from outside the community in question (n = 44/47).

Some language pairs have multiple respondents who collaborated together in responding to the questionnaire. In such cases at least one of the respondents fits the above mentioned criteria. The other respondents may be academics of unrelated fields who are community members of the language pair in question, or students working alongside the primary respondent.

Respondent selection was not randomised. The goal was to produce a global sample of language contact scenarios, which required a controlled selection of language families and areas of the world. Research coverage of language communities across the globe is skewed towards the larger world languages. Most numerically small and endangered communities lack basic linguistic descriptions^10^, let alone sociolinguistic descriptions^11^. Small language communities may only have a couple of experts who have direct experience with the communities. In some cases, the language community may no longer have an active researcher. The integrity of the sample was prioritised over respondent randomisation, ergo respondents were selected to fulfil the sampling criteria.

### Identification of explanatory factors of language change and the questionnaire design

The data for each contact pair were collected through a questionnaire designed specifically to investigate social contact. There are two parts to the questionnaire; the Overview Questionnaire investigates macro-societal and sociolinguistic characteristics mostly about the Focus language group, while the Domains Questionnaire investigates social aspects of contact across the two language communities from the perspective of the Focus group.

The Overview Questionnaire provides broad characteristics of the Focus language group as a single society. There are a range of features such as total speaker population of the Focus language, dominant subsistence patterns, and levels of jurisdictional hierarchy. Some of these features overlap with those found in the Human Relations Area File^12,13^. The Overview Questionnaire has 34 items, of which nine indicate the level of respondent confidence concerning certain questions in the Domains Questionnaire.

The Domains Questionnaire circumscribes six social domains that are typically associated with differential linguistic behaviours. The questionnaire asks a set of questions about social interaction between Focus and Neighbour group peoples across all six domains.

The sets of questions are based on groups of factors that we identified and defined based on an ongoing review of the literature. The goal of the review was to summarise various studies which suggest explanations for contact-induced change outcomes. For every source reviewed, we noted the information presented, and then evaluated any explanatory factors stated or implied in the study. By doing so we assessed the suitability of the source for investigating our overall research question.

The result of this literature review is four broad groups of explanatory factors of contact-induced change: 1) Cognitive Processes, 2) Interactions between Individuals, 3) Social Networks, and 4) Macro-contexts of Language Use. Two publications describe these explanatory factors for a linguistically oriented readership^14,15^. The general definitions are as follows:

1. Cognitive Processes: Explanatory factors that rest on general cognitive abilities at the level of the individual, such as memory, categorisation, pattern recognition, and perceptual saliency. These cognitive abilities affect language learning, production, and perception. Explanations of language contact effects assume there is a divide between native and non-native language learning and processing. Studies often focus on how one’s first language (L1) affects patterns and structures in learning a second language (L2). The age of acquisition is also thought to affect learning and adaptation abilities, such that younger children have an advantage of mastering certain kinds of linguistic structures (i.e., the “critical age” threshold).
2. Social Cognition: Explanatory factors related to individual behaviours in social interaction. These behaviours include abilities such as inferring intentions and goals of interlocutors (i.e., Theory of Mind), and the ability to adapt behaviours based on situational inferences (e.g., audience design).
3. Social Networks: Explanatory factors related to relationships and information transmission. As Social Network Theories posit, structures of relationship networks can affect how and to whom information is transmitted across a group. Social network structures refer to, for example, how many people are in a network (the population), how many relationships there are between people in any given network, the quality of these relationships, and the frequency of interaction between people in a network.
4. Macro-contexts of language use: Explanatory factors related to broader societal contexts of language use. The factors include demographic balances in an area, societal features related to history, economics, and politics. Language specific factors such as orthography and literacy, language ideologies and attitudes.

The Domains Questionnaire elicits responses to questions related to these four explanatory factors. The titular notion of domain probabilistically circumscribes social contexts of activity, role-relationships, and topics of communication^16,17^. The six social domains are Local Community, Trade, Social Exchange & Marriage, Family & Kin, Knowledge, and Labour. The domains were defined flexibly, so they may be applicable to a range of social groups. The definition and rationale for each domain can be found at https://gramadapt.clld.org/rationales.

### The collection of data by questionnaires

Respondents filled out the Domains Questionnaire first, followed by the Overview Questionnaire. The order of the domains within the Domains Questionnaire was randomised per respondent. The respondents were asked to provide the best-to-their-knowledge assessment to each question. Since the respondents were providing their subjective assessments of a language community, no personal data of individual language speakers were collected through the questionnaire.

Five questionnaires cover six social domains. Each domain questionnaire begins by asking the respondent to assess whether social contact occurs or has occurred in the domain. The respondent only continues answering the questionnaire if this is a relevant contact domain for the given language pair. If the domain is assessed as a no-contact domain, the respondent continues on to the next questionnaire. The Domains Questionnaire is completed once this process has been done for all six social domains. For each contact domain, the respondent is first asked to determine the approximate time periods of social contact, and which varieties of the Focus Language they are responding about.

Respondents filled out the Overview Questionnaire once the Domains Questionnaire was completed. As mentioned in the above section, the Overview Questionnaire asks respondents to answer questions mostly about the Focus Group as a single society. It also asks about respondents’ confidence regarding the domains and the questions asked within the Domains Questionnaire. Once the Overview Questionnaire was completed, the questionnaire ended.

The vast majority of questions in the Overview and Domains Questionnaires have a selection of predetermined answers per question from which the respondent must choose. There are three types of predetermined answer formats: a binary “yes/no” response, a Likert scale response of 1–5, and a set of partially-independent category responses. Qualitative answers were also solicited in specific parts of both questionnaires. These response types will be discussed further in the Data Records section.

The questionnaire was administered online via the survey platform Webropol. At the onset of the project in 2019 the plan was to administer the questionnaire in person by the first three authors to reduce the risk of misinterpreting the questions. The COVID-19 Pandemic required a change in approach. Respondents were asked to fill out the questionnaire within six weeks of receiving the links to their personalised questionnaires. Steps were taken to minimise any misunderstandings of the online version of the questionnaire by offering a range of support including the provision of specific instructions with examples and definitions per question, offering online video consultations, and the creation of an online chat. The uptake of support differed across respondents.

The online questionnaire format aided the reduction of certain human errors. For example, the online format allowed respondents to name and identify their Focus and Neighbour languages, which would then auto-populate the questions. The online questionnaire format also embedded automatic rules for skipping certain questions based on the previous answers chosen by the respondent. For example, when answering a question about children’s presence in a given domain, if the respondent answered that children are not present, the following questions concerning details about children’s involvement were automatically skipped.

The online format provides a visually clean administration of questions rather than a paper format, spread sheet, or document resembling a paper format. This digital format could also add information overlays to the online interface, including definitions that could be viewed by respondents by hovering their cursor over various answer options. This lessened the need to navigate between the questionnaire and support materials which were also available on a separate website. These overlays were added throughout the questionnaire to further minimise possibilities of misunderstandings.

After the questionnaire was completed, authors 1 and 3 made initial checks for answer reliability and follow ups. Once data collection for all pairs were complete, authors 1, 2 and 3 went over each respondent’s answers again to check for unclear or unusual responses. The issue of reliability judgments will be covered further in the technical validation section.

### Formatting the dataset in CLDF for distribution

Since the dataset contains information about languages, it is meant to be used (among others) by linguists and could be used together with other sets of linguistic data. We thus chose CLDF^18^ as a distribution format. In addition to interoperability, this format also provides another layer of data validation and built-in consistency checks. Finally, it allows inclusion of documentation in the dataset in a transparent way, such as the rationales informing the questionnaire questions.

The aggregation of the raw data (i.e., the questionnaire, the language metadata, the documentation and the responses) into a CLDF dataset was done using the cldfbench package^19^. Thus, CLDF creation is implemented reproducibly and can be re-run, e.g., to fix errata or update language metadata extracted from Glottolog^20^. The CLDF creation code as well as the raw data and the output CLDF data are curated in a version-controlled repository on GitHub and released versions are deposited on Zenodo for FAIR^21^ long-term access.

## Data Records

The dataset containing the responses from all 45 experts is available in CLDF format^18^ under an open license on Zenodo^22^. Some data points are anonymised at the request of the participating respondents. Further details on this are given in the Usage Notes section below.

### Data model

**Fig. 2:**
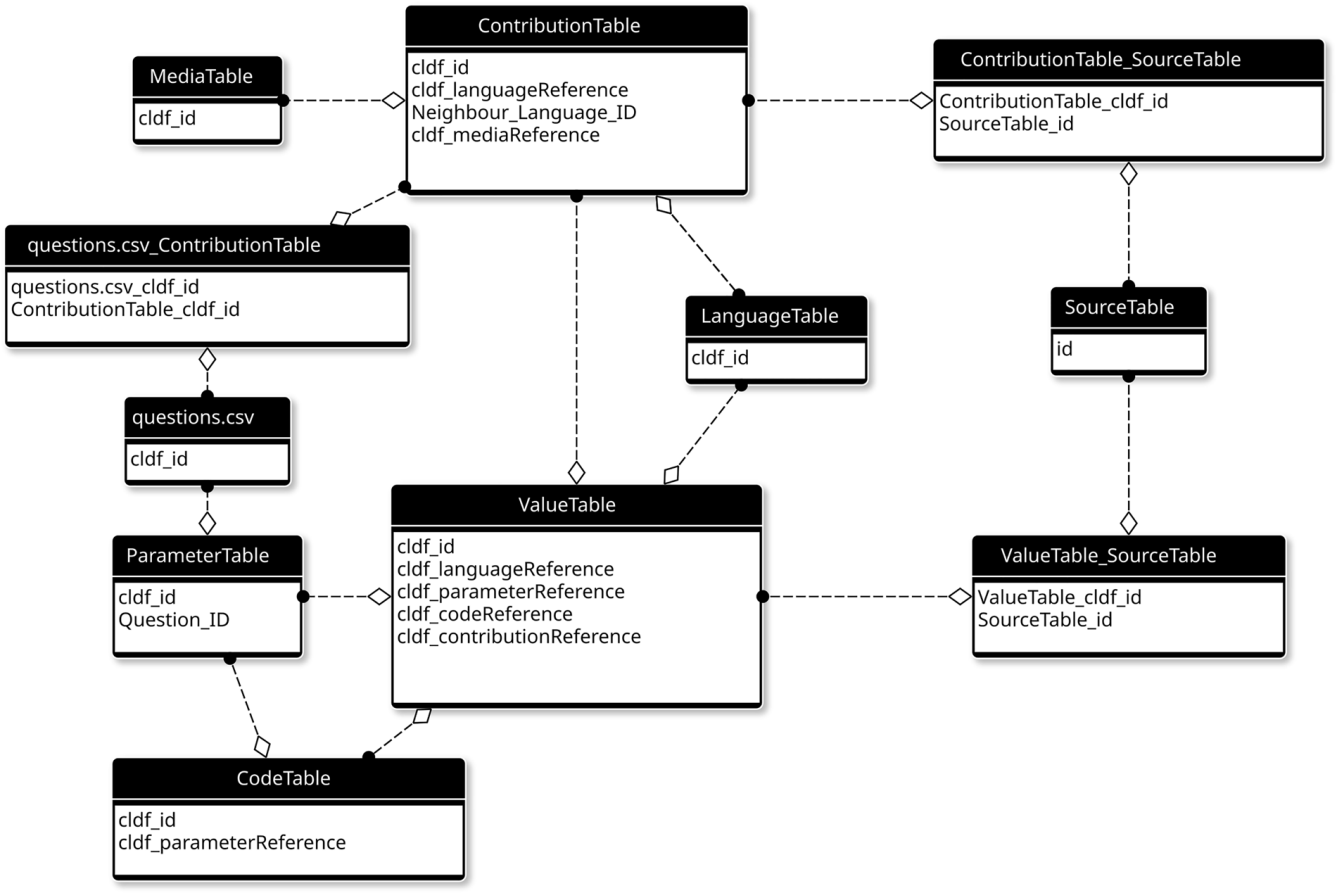
Entity-relationship diagram of the GramAdapt data model. This diagram represents the schema of the SQLite database the dataset has been loaded into using the cldf createdb command from the pycldf package. Responses are rows in the central *ValueTable*, which are linked to sources (in *SourceTable* via the association table *ValueTable_SourceTable*), (sub)questions (*ParameterTable*), response sets (*ContributionTable*) and the focus language of the respective contact pair (*LanguageTable*).

We chose CLDF as distribution format because it is particularly suitable for complex tabular data linked to languages. Following recommendations for “tidy data”^23,24^, we adopt a multi-table data model with identifiers for each row, tied together by metadata, which enables automated validation (see Fig. 2). (In the following we will use the CLDF component names for standard tables in a CLDF dataset, e.g., *LanguageTable*, and the CSV file names for non-standard tables, e.g., questions.csv).

Individual GramAdapt questions are modelled using a *ParameterTable* component and responses using a *ValueTable* – so the whole dataset fits the specification of a CLDF *StructureDataset*. CLDF datasets typically link observations to one language – not pairs of languages. Thus, we used the Focus languages as primary languages of the dataset. Focus and Neighbour languages are linked together in response sets, which we model with a *ContributionTable* – the appropriate data type for data with shared provenance. The *ContributionTable* also stores information about the other “citable units” of the dataset, i.e., the documents describing the rationale for the questionnaire construction. To distinguish between the two, a column *Type* is added to *ContributionTable*. Thus, the *ContributionTable* contains 35 rows for the response sets, marked with *Type* “contactset” and identifers of the form set[0-9]2(a|b)? (where *a* and *b* are used to disambiguate the two response sets for the same contact pair) and for the 90 rationales. The GramAdapt questionnaire sometimes groups questions together. This grouping is modelled with a custom table questions.csv and foreign keys in *ParameterTable*, linking individual questions to a question group.

CLDF datasets encode metadata (also about schema objects like tables and columns) in machine-readable form. Thus, a detailed description for human consumption can be created automatically and is available at https://github.com/cldf-datasets/gramadapt/tree/main/cldf#readme.

### The questionnaire

The GramAdapt questionnaire consists of a set of grouped questions together with rationales. As described above, this data is available from the dataset’s *ParameterTable* (and linked tables). Questions are categorised by social domain (in the *Domain* column) with the additional pseudo-domain “OV” for “overview”.

The GramAdapt questionnaire contains questions with types of responses falling into the following categories (marked with corresponding values for the *datatype* column in *ParameterTable*):

- **Binary-YesNo** A binary answer of either “Yes” or “No”.
- **Scalar** A Likert 5 point scale. The response is in textual form, but can be placed on a scale of 1–5 (e.g., “Neither positive nor negative” = 3).
- **Types** A list of preset answers where only one (or more) can be chosen (e.g., “Focus Language, Neighbour Language, Some other language, This is highly contextual”)
- **Comment** A free, textual answer.
- **Value** A numeric value.

In addition to responses conforming to one of the datatypes described above, “missing data” could be reported in two ways: A response of “B” for “blank” could be used to mark the question as relevant to the domain in question, but the respondent chose not to answer the question. This is qualitatively different from an “NA” response (“not applicable”), which indicates the question is not applicable for a particular sample set. NAs only arise in the dataset if a social domain is assessed as a “no social contact” domain by the respondent, and/or if a “no” response to questions O1, I1, or T1, renders subsequently dependent questions irrelevant (e.g., a “no” response to O1 means questions O2 and O3 will be NA).

The GramAdapt questionnaire provides documents with rationales for the questions, which often reference objects in the dataset such as other questions or external sources. We thus formatted the rationales using CLDF Markdown – a variant of markdown which allows formatting references to dataset objects using the respective object identifiers in markdown links. The CLDF Markdown is stored in the *Description* column of *ContributionTable*. For convenience, we also provide rendered versions of the rationales in the cldf/rationale/ directory of the repository.

While the domain assignment provides the major categorisation of questions, there are also several cross-cutting aspects that group questions in ways that are useful for analysis. 1) Often, similar questions are asked for each domain, e.g., whether Focus group people simplify their language when speaking in a particular domain. Such groupings can be inferred from similar formulations of the questions. 2) In addition, questions are tagged by topic (the column *Tag* in the *ParameterTable*), using the following abbreviations: P = Preamble; D = Domain characterisation; S = Social network; B = Behaviour affecting biases; O = Linguistic output of Focus group people; I = Linguistic input of Focus group people, i.e., the output of Neighbour group people; T = Language transmission to children; E = Ending questions about data source and confidence. Overview questions are tagged using OB = Behaviour affecting biases; OD = Demographics; OG = Language geography; OI = Language and identity; OL = Literacy; OS = Social structure; OH = History; OT = Time frame; OE = Respondent fieldwork experience; OC = Response confidence. 3) Finally, some questions are annotated as being part of “multi-causal factors”. In other words, we identify a number of questions in the questionnaire which represent a causal element that makes up a multi-causal one, making it possible to investigate multi-causal factors within the dataset. The following multi-causal factors are represented by columns in the *ParameterTable*:

- **Use Equivalence:** Questions pertaining to whether language use patterns are equivalent between Focus and Neighbour groups. The tag relates to questions of language use in social domains, and discrepancies in fluency between Focus and Neighbour group people when speaking.
- **Socio-Political Power:** Questions on whether there are differences in socio-political power between the Focus and Neighbour groups.
- **Language Loyalty:** For the purpose of this dataset language loyalty is defined as a tendency to be loyal to one’s language group, typically by expressing a desire to retain an identity that is expressed through the use of that language. This multicausal variable thus significantly overlaps with “Use Equivalence”.
- **Attitudes and Ideologies:** Concerns the two groups’ attitudes towards each other in general.

### The Responses

Responses to the questions in the questionnaire are modeled as rows in the *ValueTable*. These constitute the “datapoints” of the GramAdapt *StructureDataset*, i.e., they relate each answer to a language – the Focus language – via the *Language_ID* column, and a parameter – the question – via the *Parameter_ID* column. The answer is given in *Value* column, and for boolean or categorical parameters linked to a description of a fixed answer in the *CodeTable* via the *Code_ID* column.

Answers may be qualified with a *Comment*, providing clarifications, elaborations, and qualifications from the respondent regarding their response. Editors may also add their comments and clarifications, enclosed in square brackets. Finally, each answer is annotated with the *Respondent*, the name(s) of collaborator(s) who answered the particular question in the questionnaire.

There are two language pairs that stand out as peculiar. The aforementioned “Sicilian - Maltese” contact pair has two sets of responses (set06a and 06b) representing two separate time slices. “Garifuna - Galibi” (set26) only contains responses for the Overview section of the questionnaire due to timing constraints experienced by the participant, which made it impossible to contribute more.

## Technical Validation

In order to establish the validity of the dataset, we assess its technical correctness, the reliability of the responses and the construction of the questionnaire. Since a CLDF dataset can be automatically loaded into a SQLite database, we conducted some of the validation experiments below using SQL code as explained at https://github.com/cldf-datasets/gramadapt/blob/main/VALIDATION.md.

### Technical correctness

A multi-table, relational dataset is only useful if its referential integrity can be guaranteed. Thanks to using CLDF as container format, just running the cldf validate command (provided with the pycldf package) on the dataset ensures that all foreign keys are valid, i.e., resolve to valid rows in the referenced table. In particular this means that categorical questions only have valid responses as answers.

### Reliability of responses

The data are based on subjective expert assessments of sociolinguistic aspects of specific language contact scenarios. Some response sets involved pairs or teams of experts (such as sets 03 and 24), while most were the responses of single individuals. The suitability of responses was based on our judgement as language comparison experts and familiarity with the literature concerning the social aspects of language contact. Consensus from other experts aside from the respondents would have been ideal, however this step was impossible since many of the sampled language communities are under-studied with only one or two active experts. Some respondents were the sole possible candidate for answering the questionnaire. As mentioned earlier, the integrity of the sample was prioritised over other points of consideration.

Each response was manually checked twice by at least one of the first three authors. The first check involved identifying contradictory or unusual responses, as well as comments from the respondent which indicated uncertainty about the question. Responses were checked again later after the initial submission. Authors 1-3 followed up with each respondent whose responses required clarification, or looked to be an error or misunderstanding of the question.

### Suitability of the questionnaire

Since this is the first dataset of this nature to be produced in the field of linguistics, we lack a baseline to compare our questionnaire responses. We argue, however, that lines of questioning for data collection were good given the relevant and plausible distribution of answers found in the dataset. We therefore demonstrate the validity of the questionnaire by describing the distribution of the data for some key variables and data types. The distribution of the data suggests a relative lack of respondent bias, and that responses are indeed capturing differences across the sampled contact pairs. For example questions P1, the first question posed in all social domains, asks whether the social domain in question has ever been a domain of social contact between Focus and Neighbour Group peoples; e.g. does trade occur between the two groups, does marriage occur between the two groups (summary in Table 1). When divided by domain, all domains have a higher “yes” response (mean = 77.5%, median = 79.4%) than the “no” responses. The domain with the highest “yes” response is the domain of trade (DTR), while the domain with the lowest “yes” response is that of social exchange and marriage (DEM). All but one of the 34 full response sets report at least half of the social domains as contact domains. 41.18% (n = 14) of the full sets respond “yes” to contact in all six social domains, while 17.65% (n = 6) have “yes” to three out of six social domains. Only Set 11 has a single “yes” response to the single domain of Local Community (summary presented in Table 2).

**Table 1:** Distribution of “yes” vs “no” responses for question P1 per social domain.

Domain	Yes (n)	No (n)	% Yes
Social Exchange (DEM)	20	14	58.82
Family & Kin (DFK)	27	7	79.41
Knowledge (DKN)	22	12	64.71
Labour (DLB)	27	7	79.41
Local Community (DLC)	30	4	88.24
Trade (DTR)	32	2	94.12

**Table 2:** Distribution of “yes” vs “no” responses for question P1 per social domain.

Number of domains with social contact	Sets	Total
All 6 domains	02, 05, 06a, 06b, 07, 09, 12, 17, 18, 21, 22, 24, 25, 30	13
5 domains	23, 27, 29	3
4 domains	03, 04, 08, 10, 13, 15, 19, 20, 28, 32	10
3 domains	01, 14, 16, 31, 33, 34	6
1 domain	11	1

There are 13,493 data points in total, of which 2,885 data points (21.38%) are NAs. While NAs are technically “not available” data, in this dataset they can be informative and be understood as “not applicable”. Again considering questions P1, there are 204 data points across six domains, with 77.45% (n = 158) reporting a “yes” response. 22.55% (n = 46) are a “no” response. For the social domains where P1 is a “no” response, the subsequent questions of that domain are rendered “NA”. The NAs indicate that a certain social domain was irrelevant to a particular language pair, since respondents are made to skip questions where social contact is absent for the language pair in question.

**Fig. 3:**
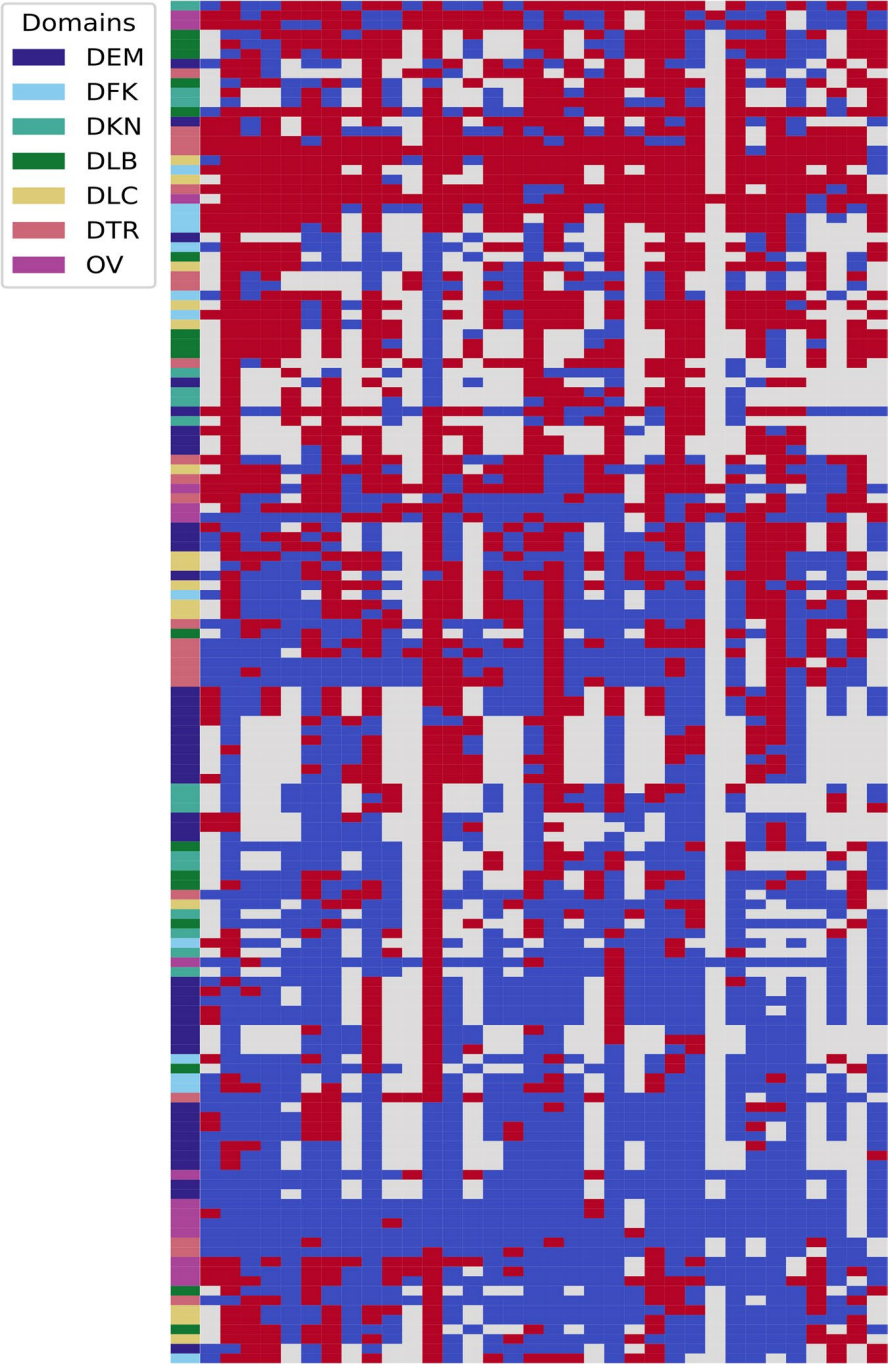
This plot shows responses for all binary questions in the dataset. Each row corresponds to one question and each column to one contact set. Responses are color-coded with red corresponding to “Yes”, blue to “No” and gray encoding missing data. Rows are clustered using a hierarchical cluster algorithm implemented in the scipy package (see https://docs.scipy.org/doc/scipy/reference/generated/scipy.cluster.hierarchy.linkage.html^25^. The first column encodes the domain each question belongs to.

We computed the distribution of responses to assess the suitability of the questions overall. For the binary questions, a heatmap can be used to visualise clusters of responses (see Fig. 3). The red cells indicate a “Yes” response, blue a “No” response, and grey an NA. While some questions from the same domain cluster closely, suggesting a lack of distinctiveness in responses, the clustering is often due to (systematically) missing data for sets where a given domain is **not** relevant. The figure shows that no two questions have identical sets of answers, suggesting the responses are sufficient across the entire dataset.

**Fig. 4:**
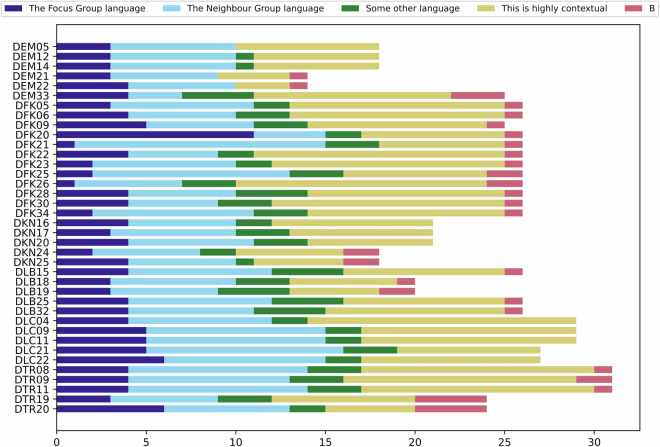
The plot shows the responses for 38 categorical questions asking for patterns of language choice in a given context. The number of responses per question is largely determined by the number of contact sets for which the corresponding domain is relevant. But the distribution of response categories per question shows the suitability of the questions to distinguish contact situations.

For question of datatype *Types* where a language was to be picked, we simply visualised the distribution of responses using a bar chart (see Fig. 4). In all questions, both of the main categories – the Focus language and the Neighbour language – were picked at least once, suggesting that the question picked up meaningful diversity.

**Fig. 5:**
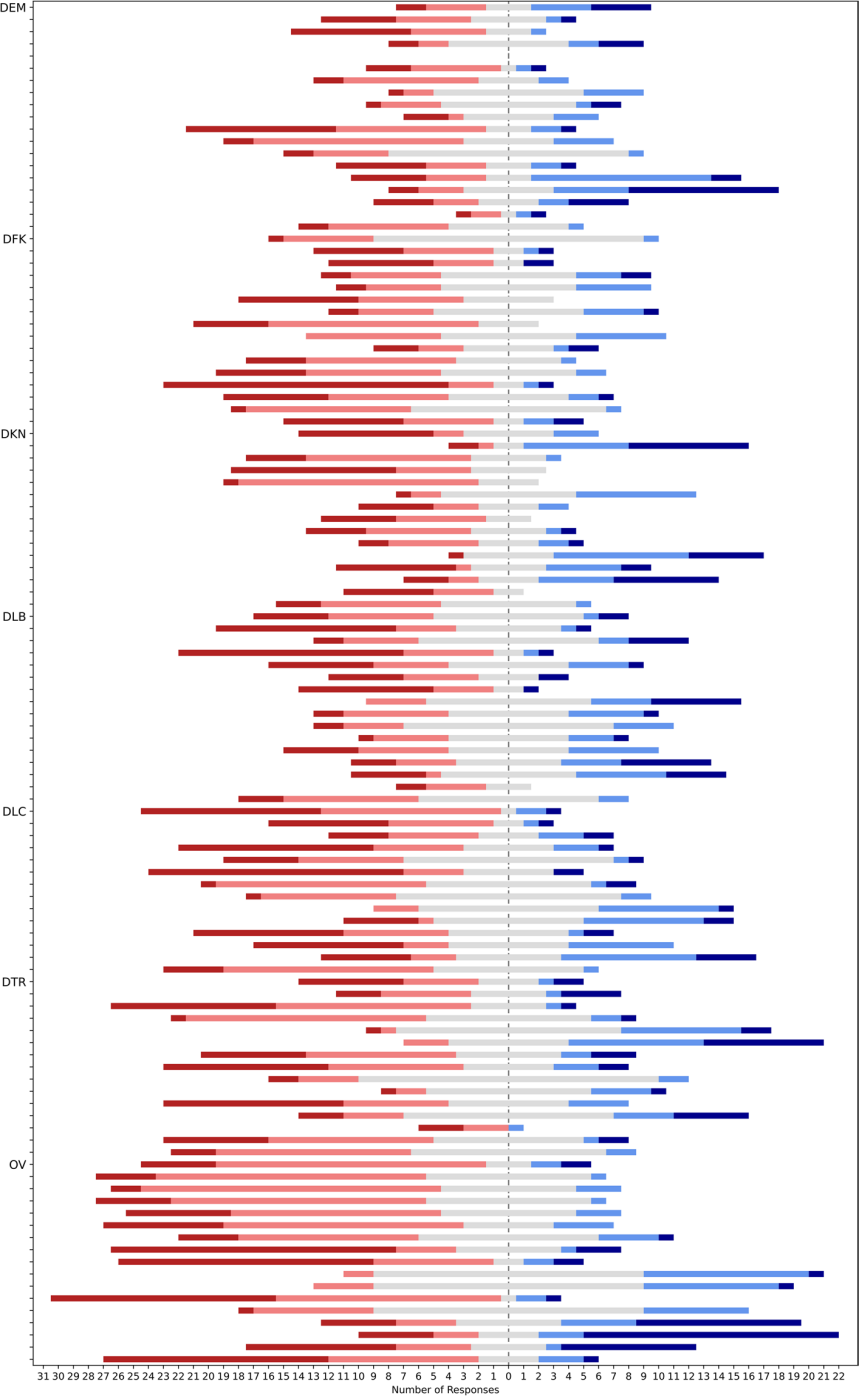
Distribution of 5-point Likert scale responses.

The last big set of questions sharing the same datatype are the questions to be answered by picking a value from a Likert scale. The majority of questions in the GramAdapt questionnaire asks for responses on a 5-point Likert scale. To visualise the distribution of responses, we plot them as diverging bar chart (Fig. 5). Just 7 out of 112 Likert-scale questions show only responses on “one side” of the scale, corroborating the suitability of the questionnaire to capture the diversity of the sampled contact sets.

## Usage Notes

The diversity of responses demonstrated in the above section shows the potential of how the dataset can be used to interrogate patterns within itself. For example, one may ask whether one social domain shows more consistency in answers across pairs compared to other domains. This question could function as a data validation step akin to the one described in the preceding section. It could also be one way to investigate an academic question, such as which contact pairs suggest consistent domain-specific contact behaviours, and which pairs are more varied.

Using the entire GramAdapt dataset quantitatively is not straightforward due to its inhomogeneous nature: Some questions have binary “yes” or “no” answers, others have answers on a Likert scale, and some have textual answers. In addition, some questions are sub-questions, or logically dependent questions meaning they cannot simply be treated in isolation. But since the GramAdapt data is available in a format that is amenable to computational re-use, exploratory analysis is easy and we can programmatically detect the issues outlined above and extract meaningful subsets of data in an informed way. Since using the GramAdapt dataset will typically mean querying and aggregating data from multiple tables, SQL — the Structured Query Language — is particularly suited for this task and since each CLDF dataset can be automatically loaded into a SQLite database (via pycldf), we recommend this approach. An overview on how to do so is given at https://github.com/cldf-datasets/gramadapt/blob/main/USAGE.md.

The CLDF format also makes it easy to load the dataset into a clld web application. Such an application is available at https://gramadapt.clld.org. It provides an entry point for qualitative analysis and a way to explore the dataset before engaging in quantitative analysis.

### Privacy

The identity of the respondent for one of the language pairs is made anonymous, and the identity of the specific languages are also obscured. The languages are represented as FL (Focus language) and NL (Neighbour language). The respondent made this request due to current and recent tensions in the area where these languages are spoken, and the potentially sensitive nature of the responses given this situation. The number of Western researchers in this area are small, hence the respondent requested that the openly available version of the dataset obscure their identity.

## Data Availability

The dataset is available on Zenodo at https://zenodo.org/records/15196305.

## Code availability

The dataset is curated in a GitHub repository (https://github.com/cldf-datasets/gramadapt) using the functionality implemented in the cldfbench package^19^. The repository includes the code to create the CLDF dataset from the “raw” data as well as the code to run the validation experiments described above (https://github.com/cldf-datasets/gramadapt/blob/main/VALIDATION.md).
